# CD57 Expression in Incidental, Clinically Manifest, and Metastatic Carcinoma of the Prostate

**DOI:** 10.1155/2014/356427

**Published:** 2014-05-22

**Authors:** Holger Wangerin, Glen Kristiansen, Thorsten Schlomm, Carsten Stephan, Sven Gunia, Annette Zimpfer, Wilko Weichert, Guido Sauter, Andreas Erbersdobler

**Affiliations:** ^1^Institute of Pathology, University Medicine Rostock, Strempelstraße 14, 18055 Rostock, Germany; ^2^Institute of Pathology, University of Bonn, Germany; ^3^Martini Clinic, University Hospital Hamburg-Eppendorf, Germany; ^4^Department of Urology, Charité University Hospital Berlin and Berlin Institute for Urologic Research, Germany; ^5^Institute of Pathology, Stendal, Germany; ^6^Institute of Pathology, University of Heidelberg, Germany; ^7^Institute of Pathology, University Hospital Hamburg-Eppendorf, Germany

## Abstract

*Objectives*. CD57 is normally found on NK-cells, but little is known about its expression in prostatic tissue. *Methods*. We investigated CD57 expression by immunohistochemistry using tissue microarrays containing 3262 prostate cancers (PCa), lymph node metastases, and benign prostatic tissue. The results were compared with clinical and pathological parameters. *Results*. Overall, 87% of PCa showed a moderate or strong expression of CD57. There was no significant difference to corresponding benign prostatic tissue. CD57 was increasingly lost from incidental over clinically manifest cancers to metastases. It correlated significantly with Gleason grade and pT-category, but not with PSA tissue expression. Loss of CD57 expression was an independent risk factor for PSA recurrence after prostatectomy in a multivariate Cox regression analysis. In standard sections, CD57 expression was heterogeneous, especially in large, high-grade PCa. *Conclusions*. There is a peculiar expression of CD57 in PCa and benign prostatic tissue. CD57 loss is associated with tumor dedifferentiation and tumor size. However, the use of this marker for prognostic purposes is hampered by its heterogeneous expression.

## 1. Introduction

CD57 is a sulfated carbohydrate chain containing the epitope for the antibody HNK-1 [1, 2]. The exact function of this molecule on the cell surface is still not completely understood [3]. It was first described on natural killer lymphocytes (NK-cells), but it is also expressed on a variety of normal and neoplastic cells, especially from the central nervous system. Among epithelial cells, only neuroendocrine cells, thyroid cells, renal tubular cells from the loop of Henle, and prostate glands are consistently positive [4]. Some neuroendocrine lung cancers and thyroid carcinomas are also positive [5, 6]. In pathology laboratories, antibodies against CD57 are routinely used to detect NK-cells or cells of neural/neuroendocrine origin. However, there exist only few reports, with a small number of patients each, on the frequency and characteristics of CD57 expression in prostate cancer [4, 7–9].

## 2. Material and Methods

### 2.1. Patients and Tissues

The study consisted of 6688 tissue specimens from 4748 patients with prostate cancer (PCa). All specimens were collected in a retrospective manner from the material that remained after the diagnostic process was completed, according to the local review board and data safety laws. Clinical (age, prostate-specific antigen values, and follow-up) and pathological (pT-classification, Gleason grade) data were collected prior to complete anonymisation of the cases, and Excel tables were created. The tissue specimens were assembled in tissue microarrays (TMAs) as previously described [10] and pooled in five different sets of TMAs as follows.

1. *TMA “incidental PCa”*: this TMA contained 59 tissue spots from 59 patients with prostate cancers incidentally detected in the material from transurethral resections performed because of benign prostatic hyperplasia (BPH). The tissue spots had a diameter of 2 mm and were combined in one paraffin block. With this TMA, the frequency of CD57 expression in incidental prostate cancers was investigated.
2. *TMA “prostatectomy 1”*: this TMA contained 2910 tissue spots from 970 patients, whose prostate cancers were treated by radical prostatectomy at the Charité Universitätsmedizin Berlin. Each patient was represented in the TMA by two different spots from the largest tumour focus in the prostate and by one spot from benign prostatic tissue. The spots had a diameter of 1.2 mm and were combined in 20 paraffin blocks. This TMA served for the comparison of CD57 expression in normal and neoplastic tissue, the overall frequency of CD57 expression in clinically manifest PCa, and the correlation with T-classification and grade.
3. *TMA “prostatectomy 2”*: this TMA contained 3261 spots from 3261 PCa patients, treated by radical prostatectomy at the University Hospital Hamburg-Eppendorf. Each patient was represented by a single spot with a diameter of 0.6 mm from the largest tumour focus. The tissue spots were combined in seven paraffin blocks. Each block additionally contained 58 control spots from various nonprostatic benign and malignant tissues. This TMA was used to validate the frequency of CD57 expression in a second patient cohort to the “prostatectomy 1” TMA and to correlate CD57 expression with pT-classification and grade. This TMA was also stained by immunohistochemistry against PSA, thus allowing for a direct case-by-case comparison of both markers. Furthermore, since follow-up data were available for the majority of these patients, a correlation of CD57 expression with serologic PSA recurrence after radical prostatectomy could be performed. PSA failure was defined as a persisting or increasing postoperative PSA value >0.1 ng/mL in 2 separate measurements.
4. *TMA “prostatectomy 3*”: this TMA contained 348 spots with a diameter of 0.6 mm, assembled in 2 blocks, from 348 PCa patients treated by radical prostatectomy. Tissues were retrieved from the archives of the Institute of Surgical Pathology, University of Zurich, Switzerland. This TMA was used for an external validation of anti-CD57 staining and evaluation and also for the correlation of CD57 expression with pT-category and Gleason grade.
5. *TMA “lymph nodes”*: this TMA contained 94 spots from regional lymph node metastases from 94 PCa patients treated by radical prostatectomy. The spots had a diameter of 1.5 mm and were combined in two paraffin blocks. With this TMA, the frequency of CD57 expression in regional lymph node metastases could be investigated. Surrounding lymphatic tissue with interspersed NK-cells served as an internal positive control.

Additionally, standard tissue sections from 16 patients with PCa were included in the study to investigate the heterogeneity of CD57 expression in PCa.

### 2.2. Immunohistochemistry

The immunohistochemical stains were performed with the avidin-biotin complex (ABC) method [11]. In the TMAs “incidental PCa,” ”prostatectomy 1,” “prostatectomy 2,” and “lymph nodes,” it worked on an automated stainer (Autostainer Link 48, DAKO, Denmark), using an anti-CD57 antibody (clone NK-1, Menarini, Berlin, Germany) at a dilution of 1 : 10 after a pretreatment with proteinase K for 10 min. For the TMA “prostatectomy 2,” immunohistochemical stains against prostate-specific antigen were already available from previous studies [12]. The TMA “prostatectomy 3” was stained on Ventana Benchmark stainer (Roche, Basel, Switzerland) with another CD57 antibody (clone TB01, DAKO) at a dilution of 1 : 50.

### 2.3. Evaluation

Microscopic slides were investigated by Glen Kristiansen (TMA “prostatectomy 3”) and Holger Wangerin and Andreas Erbersdobler (other 4 TMAs). Evaluation of CD57 and PSA expression was scored in a semiquantitative fashion as previously described [12, 13]:

- score 0: completely negative staining;
- score 1: staining intensity weak or heterogeneous in <50% of cells;
- score 2: staining intensity moderate or heterogeneous in >50% of cells;
- score 3: staining intensity strong in all or near all cells.

For some statistical comparisons, scores 0 and 1 and scores 2 and 3 were grouped together as “low” and “high,” respectively.

The 16 standard sections were evaluated for heterogeneous staining by investigating several ocular fields with a 20x objective (microscope Axiostar Plus, Zeiss, Oberkochen, Germany), representing an area of 0.97 mm², and assigning a score to these “tissue spots” as described above.

### 2.4. Statistics

Statistical calculations were performed with the software packages SPSS, version 20.0.0 (IBM Corp., NY, USA), and PRISM, version 2.01 (GraphPad, CA, USA). The *χ*²-test, Fisher's exact test, and Spearman's rho were used to calculate correlations between variables. Kaplan-Meier curves, the log-rank test, and Cox proportional hazards regression were used to compare PSA recurrence rates.

## 3. Results

**Figure 1:**
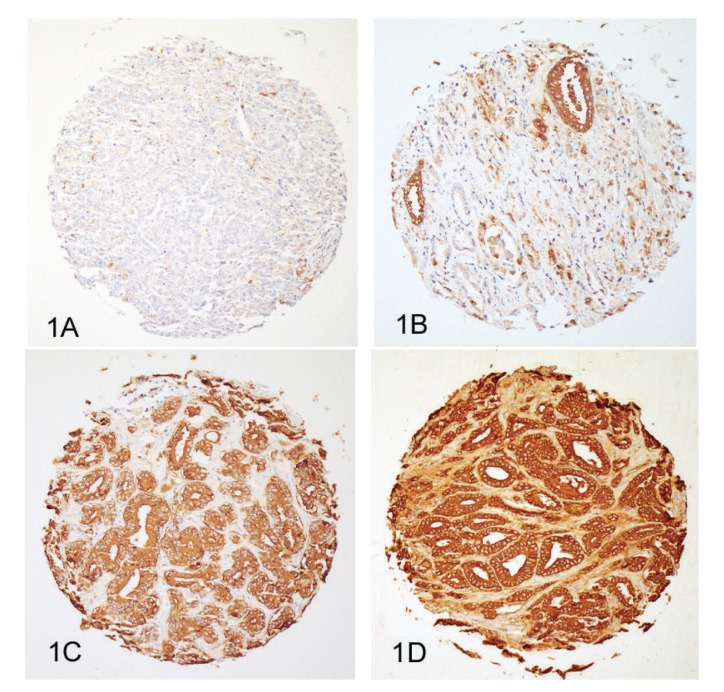
Prostate cancers from the TMA “prostatectomy 2” with different expression levels of CD57. (A) Absent expression (score 0). (B) Weak expression (score 1). (C) Moderate expression (score 2). (D) Strong expression (score 3). Immunohistochemistry with an antibody against CD57; original diameter of the TMA spot: 0.6 mm.

**Figure 2:**
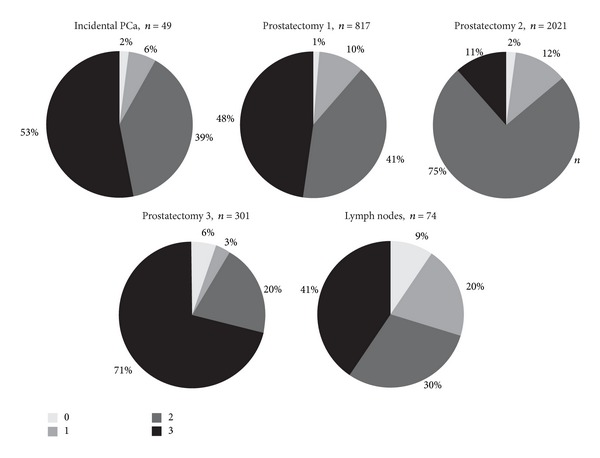
Frequency of CD57 staining scores in prostate cancers from five different TMAs expressed as percentage. Each circle diagram depicts one TMA. The staining scores from 0 to 3 correspond to the different sections from light-grey (score 0) to black (score 3).

The clinical and pathological characteristics of the patients in the respective cohorts are presented in Table 1. Overall, 3262 PCa could be evaluated for CD57 expression after immunostaining of the five TMAs. Missing cases resulted from spots that floated off during the staining procedure or were due to the absence of cancer cells in the tumor area. Immunostaining usually showed a strong and distinctive membranous and cytoplasmic positivity in epithelial cells and lymphocyte subpopulations and an absence in stromal cells. Examples of the four different staining intensities are presented in Figures 1(A)–1(D). A weak or absent expression of CD57 was found in 4 cases (8%) from the TMA “incidental PCa,” in 93 cases (11%) from the TMA “prostatectomy 1,” in 281 cases (14%) from the TMA “prostatectomy 2,” in 27 cases (9%) from the TMA “prostatectomy 3,” and in 22 cases (29%) from the TMA “lymph nodes.” Details of the proportions of staining patterns in the five TMAs are given in Figure 2.

The TMA “prostatectomy 2” allowed for a comparison between tumor and benign tissue in individual cases. In the majority of cases (90%), staining intensities differed by no more than one scoring point. Only seven cases (1%) showed a significant stronger CD57 expression in the tumor, whereas 70 cases (9%) displayed a weaker expression by more than one scoring point as compared to the corresponding benign prostatic tissue.

**Figure 3:**
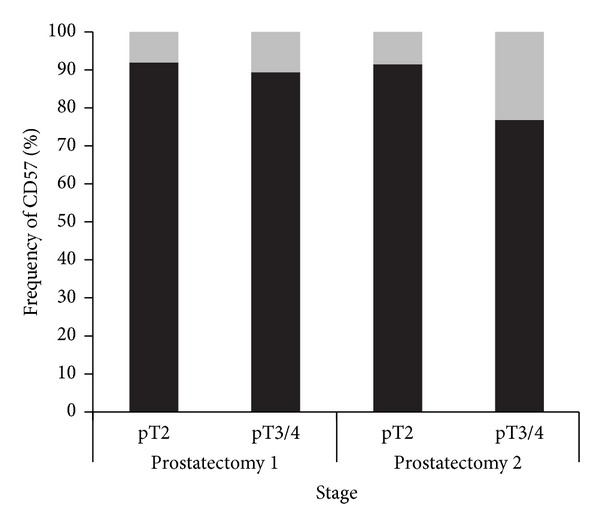
Correlation of CD57 expression in the TMAs “prostatectomy 1 and prostatectomy 2” with T-classification. T-classification and staining patterns were dichotomised into the prognostically relevant categories pT2 versus pT3/4 and absent/low (grey) and moderate/strong (black) CD57 staining, respectively. Differences are statistically significant with the *χ*²-test in the TMA “prostatectomy 2” (*P* < 0.0001), but not in the TMA “prostatectomy 1” (*P* = 0.307).

**Figure 4:**
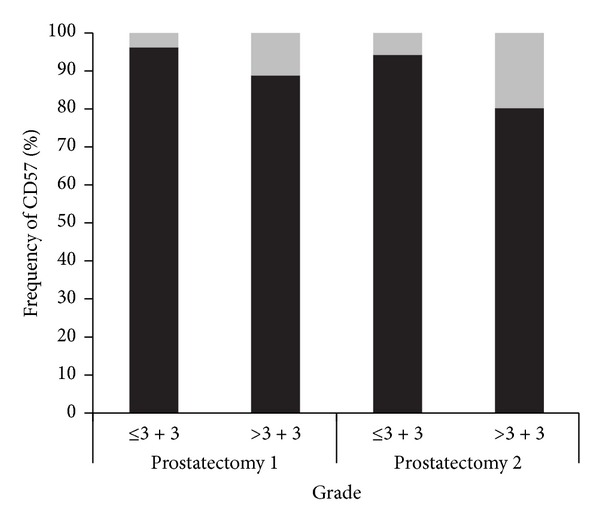
Correlation of CD57 expression in the TMAs “prostatectomy 1 and prostatectomy 2” with Gleason grade. Gleason grades and staining patterns were dichotomised into presence or absence of a Gleason 4 pattern and absent/low (grey) and moderate/strong (black) CD57 staining, respectively. Differences are statistically significant with the *χ*²-test in both TMAs (*P* < 0.0001 and *P* = 0.0032, resp.).

Comparisons between CD57 expression and tumor stage and grade were performed in the three prostatectomy TMAs. For statistical calculations in the comparisons of the first two TMAs, staining patterns and pathological tumor characteristics were dichotomised. The correlation of an absent/low CD57 expression with a pT3/4 stage was statistically significant in the TMA “prostatectomy 2” (*P* < 0.0001), but not in the TMA “prostatectomy 1” (*P* = 0.307). In both TMAs, a statistically significant correlation of an absent/low CD57 expression and a Gleason score ≥3 + 4 was present (*P* < 0.0001 and *P* = 0.0032, resp.). Figures 3 and 4 illustrate these comparisons. In the TMA “prostatectomy 3,” Spearman's rho was used to evaluate a correlation of the discrete variables. It showed a significant correlation coefficient between CD57 expression and pT-category (0.179; *P* = 0.002) as well as with Gleason grades (0.234; *P* < 0.0001).

**Figure 5:**
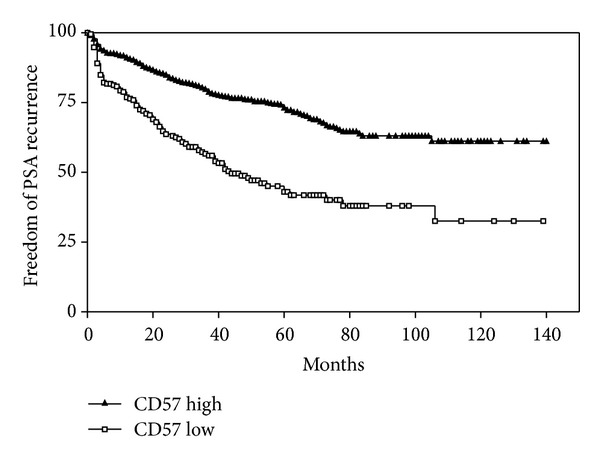
Kaplan-Meier curve showing freedom from PSA recurrence in the TMA “prostatectomy 2” (1916 patients), depending on high (staining scores 2 and 3) or low (staining scores 0 and 1) expression of CD57 in prostate cancer cells. Differences between curves are statistically significant with the log-rank test (*P* < 0.0001).

In the TMA “prostatectomy 2,” follow-up data on serologic PSA values after prostatectomy were available in 1916 patients, with a follow-up period of 1 to 144 months (mean, 34 months). In Kaplan-Meier curves, an absent/low expression of CD57 showed a greater likelihood of PSA failure after radical prostatectomy (Figure 5). In a univariate analysis using the log-rank test, the difference of the survival curves was statistically significant (*P* < 0.0001). In the Cox proportional hazards regression, CD57 expression remained an independent parameter after the stepwise inclusion of the variables pT-category, Gleason grade, and margin. The results of the Cox analysis are given in Table 2.

**Figure 6:**
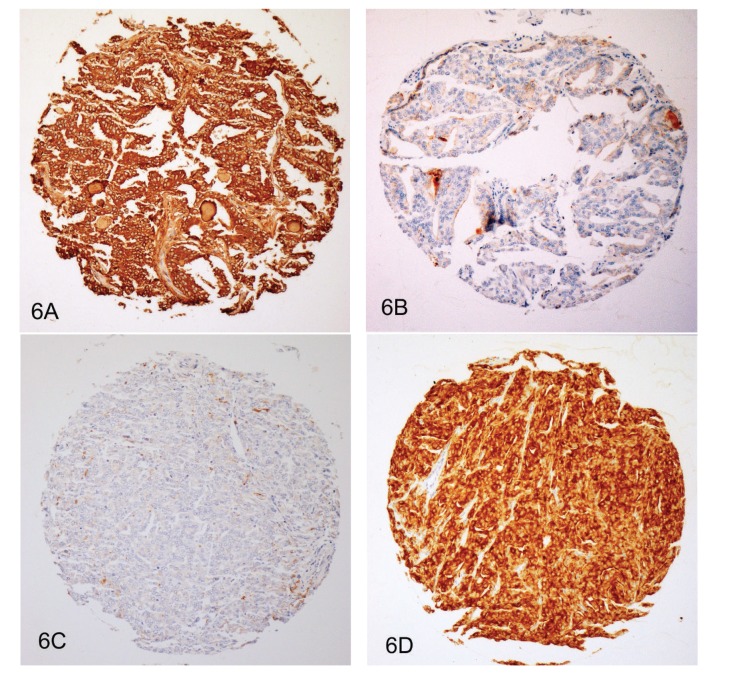
Two prostate cancers from the TMA “prostatectomy 2” with markedly different expression levels of CD57 and PSA. (A) Prostate cancer with a strong expression of CD57. (B) Same case (deeper section) as (A) with a nearly absent expression of PSA. (C) Prostate cancer with an absent expression of CD57. (D) Same case (deeper section) as (C) with a strong expression of PSA. Immunohistochemistry with antibodies against CD57 and PSA; original diameter of the TMA spot: 0.6 mm.

Since the TMA “prostatectomy 2” was also stained by immunohistochemistry against PSA, a direct comparison of CD57 and PSA in the same tumors, using the same scoring system, was possible for 1672 cases. Among these, 1346 (80%) differed by no more than one scoring point. There were, however, 198 cases (12%) with high PSA expression (score 2 or 3), but low CD57 expression (score 0 or 1), and 128 cases (8%) with the reverse staining pattern. Two prostate cancers with markedly different staining patterns of CD57 and PSA are shown in Figure 6.

**Figure 7:**
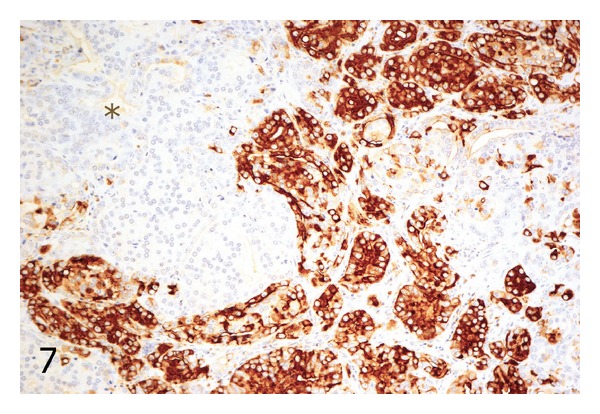
Standard histologic section showing a prostate cancer with heterogeneous expression of CD57. The area with an asterisk shows a complete absence of CD57 staining, whereas adjacent tumor glands in the upper right and lower middle demonstrate a strong staining pattern. Immunohistochemistry with an antibody against CD57; original magnification ×100.

**Figure 8:**
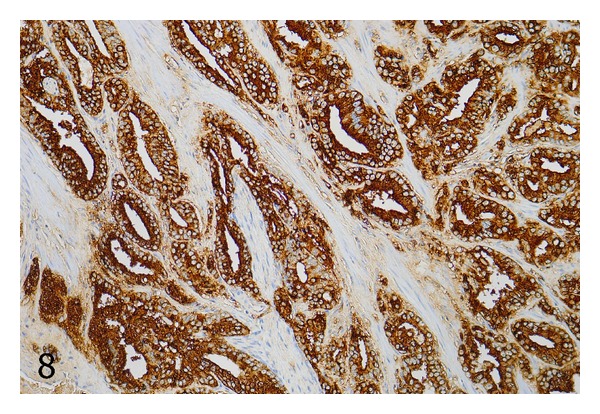
Standard histologic section showing a prostate cancer with homogeneous expression of CD57. Immunohistochemistry with an antibody against CD57; original magnification ×100.

Additionally, 16 standard sections containing prostate cancers from 16 radical prostatectomy specimens were investigated for heterogeneity of CD57 staining. We used ocular fields in between the size of the spots from the prostatectomy TMAs and assigned staining scores to each field in the tumor area. By this method, nine cases contained fields that differed in their CD57 expression by no more than one scoring point, whereas the other seven cases showed ocular fields that differed by more than one scoring point.  Figure 7 illustrates a PCa from a standard section with a heterogeneous expression of CD57, and Figure 8 shows a case with a homogeneous staining pattern.

## 4. Discussion

In 1985, Rusthoven et al. investigated a bone marrow specimen by immunohistochemistry against CD57 and incidentally found a positive staining in metastatic prostate cancer cells. Three resection specimens from benign prostatic hyperplasia (BPH) were also positive for CD57 [14]. In the same year, Wahab and Wright investigated 60 specimens containing PCa, normal prostate tissue, and BPH for CD57 expression. They found the strongest expression in BPH tissue and well to moderately differentiated cancers. All PCa with the exception of two high-grade cases were positive for CD57 [4]. This was confirmed in 2 studies from 1987 and 1989 with 21 and 28 cases, respectively [7, 8].

McNeal et al. described a CD57 expression in 15 cases of prostatic intraepithelial neoplasia (PIN) [15]. The largest study so far came from Liu et al. in 1995. They investigated 52 cases of prostate cancer for CD57 expression. These authors were the first ones to describe a statistical correlation between CD57 expression and tumor grade and stage [9].

For nearly two decades, no further studies have focussed on the topic of CD57 expression in adenocarcinoma of the prostate. However, immunohistochemical techniques have improved significantly since then. Antibodies against CD57 nowadays are routinely established in pathology laboratories, working well in automated staining systems. The tissue microarray (TMA) technique, despite its well-known drawbacks, allows for a simultaneous and cost-effective immunohistochemical investigation of hundreds and thousands of tumor specimens [16]. Carcinoma of the prostate, the most prevalent malignant tumor of human males, is among the most intensively studied tumors with regard to tissue markers [17, 18]. However, the fact that prostate cancer shows a particular expression of CD57 seems to have been nearly buried into oblivion among the society of pathologists.

We used five different TMAs for a comprehensive analysis of CD57 expression. The TMA “incidental PCa” is assumed to contain mostly indolent tumors, whereas the TMA “lymph nodes” were made of metastases, thus representing aggressive cancers. The three “prostatectomy” TMAs, as a matter of fact, contained a collection of tumors with different stages and grades. Since these three TMAs were derived from three different prostate cancer centers, we were to exclude any bias concerning patient selection, specimen fixation, and paraffin embedding.

We could confirm the high rate of CD57 expression in prostate cancers. Eighty-seven percent of all tumors investigated in our TMAs were moderately or strongly positive for this marker. The results from the TMA “prostatectomy 3” proved that this effect was also found when immunohistochemical staining was performed with a different antibody on a different staining automat, in a different laboratory, and evaluated by a different observer. It therefore served as an extern validation to the staining patterns of the other TMAs.

A comparison with corresponding benign prostatic tissue showed that CD57 expression in the prostate is not a tumor-specific phenomenon. The function of CD57 in prostatic epithelial cells is still not known. A role of this protein in the mediation of cell contacts, especially in the nervous system, is debated [19], but it is unclear why this should take place in the prostate, but not in most other epithelial cells. By immunohistochemical comparisons, we were able to show that the CD57 expression, despite of its frequency, is not in all cases congruent to the expression of PSA, the standard marker of prostatic derivation. Thus, CD57 expression does not seem to be a function of PSA production in prostate cells.

There was a stepwise increase in the fraction of cases with a CD57 loss from the TMA “incidental PCa,” over the cancers in the three “prostatectomy” TMAs, to the “lymph nodes” TMA. Whereas the first and the latter by definition represent tumors of different stages and aggressiveness, the cancers in the three “prostatectomy” TMAs are probably more heterogeneous in their biologic behaviour. In the “prostatectomy” TMAs, there was a significant association of CD57 staining with stage and Gleason grade. Consequently, tumors with an absent or low CD57 expression showed a higher rate of PSA recurrences in a univariate analysis on Kaplan-Meier curves and in a multivariate Cox regression with the parameters pT-classification, Gleason grade, and margin. In the standard sections, it became evident that CD57 expression is often heterogeneous, especially in larger tumors with higher Gleason grades.

Thus, from the results of the present study and the sparse literature on that topic, it seems that CD57 is expressed in a high proportion of benign prostatic tissue, PIN, and early, low-grade PCa. With tumor growth and/or the evolution of high-grade clones, CD57 expression is gradually and focally lost. In TMA spots, the likelihood of having sampled a CD57 negative area is higher in larger tumors with higher Gleason grades.

## 5. Conclusions

Pathologists, clinicians, or patients can take advantage from the information provided by our study as follows.

First, CD57 may be used as a marker of prostatic derivation in equivocal cases, such as tumors from the prostatic urethra or bladder neck, or in metastatic cancers of unknown primary (CUP). Beside prostate cancer, only neuroendocrine lung cancers and thyroid cancer frequently express CD57, whereas the expression in other cancer types is rare and usually weak [5–7]. Beside PSA, the marker most commonly used, there have been several more or less specific immunohistochemical markers for prostatic tissue reported [18]. But not all of these markers are established in every pathology laboratory, and sometimes it is desirable to rely on more than one or two markers.

Second, CD57 is not useful to distinguish between benign and malignant prostatic tissues.

Third, reduced or absent CD57 expression can be regarded as a prognostic marker and may help to stratify patients in different risk groups. However, our results indicate that loss of CD57 seems to occur in a heterogeneous fashion in larger tumors. Complete loss of CD57 expression is a rather infrequent event in clinically manifest tumours and even in metastases. Nevertheless, in individual cases, CD57 may be of some prognostic value in biopsy specimens.

Fourth, the strong expression of CD57 raises the question whether this molecule may serve as a target for new therapeutic strategies in PCa. However, the fact that CD57 is also normally expressed in other tissues, especially in neural tissue, is an argument against its use as a therapeutic target because significant side effects would have to be feared.

Pathologists should be familiar with the peculiar expression of CD57 in benign and malignant prostatic tissue and with the diagnostic and prognostic potential of this marker.

## Figures and Tables

**Table 1:** Clinical and pathological data of patients in TMAs.

TMA	Number of cases	Years of tissue collection	Median patient age (years)	PSA (ng/mL)	T-classification*	Gleason ≤3 + 3*	Gleason ≥3 + 4*
Incidental PCa	59	1995–2007	66	9.26 (median)	T1a: 32 (54%) T1b: 27 (46%)	50 (85%)	9 (15%)
Prostatectomy 1	970	1999–2007	62	8.48 (median)	pT2: 509 (69%) pT3/4: 224 (31%)	239 (33%)	492 (67%)
Prostatectomy 2	3261	1992–2004	62	9.06 (median)	pT2: 2080 (67%) pT3/4: 1023 (33%)	1426 (46%)	1679 (54%)
Prostatectomy 3	348	n.a.	64	<10: 136 (45%) ≥10: 168 (55%)	pT2: 220 (64%) pT3/4: 125 (36%)	56 (16%)	290 (84%)
Lymph nodes	94	1998–2010	63	n.a.	n.a.	n.a.	n.a.

*Numbers given do not add up to numbers of cases because of lacking data in some cases.

n.a.: not available.

**Table 2:** Results of Cox regression analysis on the TMA “prostatectomy 2” (1916 patients).

	Coeff.	SE	Wald	df	*P *	HR	Low 95%	High 95%
Gleason >3 + 3 versus ≤3 + 3	1.247	0.183	46,231	1	<0.0001	**3.480**	2.429	4.985
pT3/4 versus pT2	1.146	0.133	73,789	1	<0.0001	**3.146**	2.422	4.086
Margin positive versus negative	0.653	0.108	36,332	1	<0.0001	**1.922**	1.554	2.322
CD57 high versus low	−0.486	0.115	18,532	1	<0.0001	**0.609**	0.486	0.763
